# Hidden Fungi: Combining Culture-Dependent and -Independent DNA Barcoding Reveals Inter-Plant Variation in Species Richness of Endophytic Root Fungi in *Elymus repens*

**DOI:** 10.3390/jof7060466

**Published:** 2021-06-09

**Authors:** Anna K. Høyer, Trevor R. Hodkinson

**Affiliations:** Botany, School of Natural Sciences, Trinity College Dublin, The University of Dublin, Dublin D2, Ireland; hye9ra@gmail.com

**Keywords:** DNA barcoding, *Elymus repens*, fungal root endophytes, high-throughput amplicon sequencing, MEA, PDA

## Abstract

The root endophyte community of the grass species *Elymus repens* was investigated using both a culture-dependent approach and a direct amplicon sequencing method across five sites and from individual plants. There was much heterogeneity across the five sites and among individual plants. Focusing on one site, 349 OTUs were identified by direct amplicon sequencing but only 66 OTUs were cultured. The two approaches shared ten OTUs and the majority of cultured endophytes do not overlap with the amplicon dataset. Media influenced the cultured species richness and without the inclusion of 2% MEA and full-strength MEA, approximately half of the unique OTUs would not have been isolated using only PDA. Combining both culture-dependent and -independent methods for the most accurate determination of root fungal species richness is therefore recommended. High inter-plant variation in fungal species richness was demonstrated, which highlights the need to rethink the scale at which we describe endophyte communities.

## 1. Introduction

Plants are surrounded by microorganisms living on seeds, roots, leaves and flowers [1,2,3]. Microorganisms found within asymptomatic plants, which are classified as endophytes [4,5], have gained a lot of attention from ecologists, agronomists and pharmacists. Endophytes have been shown to be able to shape the plant community [6] and their associated food webs [7]. Furthermore, some species of endophytes have been shown to provide plants with benefits such as drought tolerance [8], heat tolerance [9], salt stress tolerance [10], improved mineral nutrition [11], as well as protection against diseases [12,13] and pests [14]. In addition, useful secondary metabolites have been isolated from endophytes such as sphaeropsidin A, sphaeropsidin D and acetylsphaeropsidin A, which have shown anti-cancer properties [15].

Studies can deploy culture-dependent and/or direct sequencing methods to describe endophyte communities of plants. When studies use the culture-dependent method, the surface-sterilised tissue is placed on an agar-based medium and, once the endophyte grows out, they can be maintained in pure culture followed by identification on the basis of morphology as well as using DNA sequencing. Another possibility is to identify endophytes directly from the plant material using DNA sequencing. In this case, DNA is extracted from the surface-sterilised plant tissue and a set of primers are used in PCR to obtain sequences of interest. The obtained sequences are then compared to sequences with ‘known identity’, often but not always, using a public database [16].

It is generally accepted that not all fungi will grow on all artificial media [17] and studies have shown that different media can influence the number of isolated endophytes as well as the species richness [18,19,20]. In investigations of non-clavicipitaceous fungal endophytes of grasses, most often only one type of media is used [21,22,23] and when multiple types are used, the effect is not discussed [24,25]. In this study, the variation in species richness isolated on the three most commonly used media of non-clavicipitaceous fungal endophytes of grasses, PDA, MEA and 2% MEA, were investigated and compared.

Furthermore, it is standard practice to pool samples independent of whether the endophyte study is based on direct sequencing or culturing without taking into account inter-plant variation [26,27]. To address this, we investigated the variation at site level as well as at individual plant level in the wild and serious grass weed species *Elymus repens* and discuss whether it is reasonable to pool samples. In addition, comparisons are made between the communities estimated by direct sequencing and culturing. The results help to optimise the discovery efficiency of endophytes and better understand the factors influencing their diversity.

## 2. Materials and Methods

### 2.1. Elymus Repens Sampling

Plant collections were initiated in August 2016 from a total of five fields in Ireland. *Elymus repens* was targeted because it is a wild relative of many important cereals and the species could host compatible and beneficial root endophytes. *Elymus repens* is placed in the tribe Triticeae that also includes barley, rye, and wheat, but the relationships of taxa within this tribe are debated [28]. The perennial *Elymus repens* is native to Europe and Asia and is capable of extensive vegetative spread via rhizomes which has made it a serious weed in fields [29]. Site I was situated in Johnstown in Kildare (53.22884° N; −6.61186° W) where the present crop was barley, site II and III were at Kildalton Agricultural College in Kilkenny (52.34397° N; −7.30638° W and 52.35636° N; −7.31603° W) with barley and wheat. The last two sites, IV and V, were situated in Cork (51.81678 °N; −8.49056 °W and 51.8526° N; −8.04323° W), where the crop was winter wheat and barley. Ten plants were sampled from each field, except from site V, where only eight plants were sampled. Plants were sampled from field margins where barley or wheat had been grown and there was a record of high disease pressure from *Fusarium* spp. and take-all caused by *Gaeumannomyces graminis*. Individual plants were kept at 4 °C in their clump of soil until they could be processed.

### 2.2. Root Surface Sterilisation and Endophyte Culturing

Endophytes were isolated from roots of *Elymus repens*. Roots were washed in plenty of tap water. The cleanest roots were cut from the root system of each plant and surface sterilised. The surface sterilisation was performed in six steps. Between each step the roots were transferred to a new sterile 50 mL tube with ethanol wiped forceps. The sterilisation was performed as follows: I. 25 mL autoclaved ultrapure water (Purite Select Fusion (Thame, Oxon, UK), max. 18.2 MΩ.cm, shaken at 350 rpm for 1 min (min); II. 25 mL 70% ethanol shaken at 350 rpm for 3 min; III. 25 mL 5% sodium hypochlorite (NaOCl) shaken at 350 rpm for 10 min; IV–VI. 25 mL autoclaved ultrapure water shaken by hand for 1 min at each round. After the third washing step the roots were transferred to an empty Petri dish and cut into 2 mm long pieces. Five root pieces were placed on three types of media, potato dextrose agar (PDA), malt extract agar (MEA; standard 4.5%) and 2% MEA, and 1 min imprints of five root pieces were also made on PDA to test for possible epiphytic contamination. The surface sterilisation technique was found to be efficient in eliminating epiphytes. Samples were cultured at 25 °C for up to 35 days. Subcultures were made on the original medium.

### 2.3. High-Throughput Amplicon Sequencing

The remaining fraction of the surface-sterilised root system from individual plants was stored at −80 °C and then used to run direct amplicon sequencing of root DNA on a high-throughput Illumina paired-end sequencing platform. Individual root systems were freeze dried and each sample was then disrupted using a mixer mill (Retsch MM 300; Haan, NRW, Germany) with three surface-sterilised 4 mm glass beads at 30 freq 1/s for 5–30 min dependent on the toughness of the sample. Novogene Co. Ltd. (Cambridge, Cambs., UK) performed the DNA extraction and sequencing. Extraction used 0.1 g of root and the CTAB procedure. PCR reactions were carried out with Phusion^®^ High-Fidelity PCR Master Mix (New England Biolabs; Hitchin, Herts., UK) and used the fITS7 [30] and ITS4 [31] primers to amplify the nrITS2 region. The fITS7 primer targets a binding site in the 5.8 S region adjacent to the ITS2 spacer. In combination with the ITS4 primer, the primers yield amplicons that span the ITS2 region only. PCR products were purified with Qiagen Gel Extraction Kit (Qiagen, Hilden, NRW, Germany) and the libraries generated with NEBNext^®^ UltraTM DNA Library Prep Kit (Hitchin, Herts., UK) for Illumina and quantified via Qubit and Q-PCR. The nrITS2 DNA was sequenced for individual samples at 100,000 raw tags/sample using an Illumina PE250 platform.

### 2.4. Fungal DNA Extraction, Amplification and Sanger Sequencing

DNA for Sanger sequencing was extracted using predominately the DNeasy Plant mini Kit from Qiagen (Hilden, NRW, Germany). For samples where this procedure did not work, the NucleoSpin plant kit from Macherey-Nagel (Duren, NRW, Germany) was utilised. Independent of the kit, the subsequent steps were performed. Under sterile conditions 1/8th of fungal culture growing on a Petri plate was scraped with a sterile scalpel and put into a 1.5 mL microcentrifuge tube. A sterile metal bead was added to the tube and the sample was disrupted using a mixer mill (Retsch MM 300; Haan, NRW, Germany) for 30 s at 20 Hz. The final volume was 50 µL for both kits.

PCR was prepared for a total volume of 12.5 µL using BioMix from Bioline (London, UK). For the first 96 well plate, 0.5 µL DNA template was used and for the subsequent plates 1 µL DNA template (approximately 100 ng µL^−1^) was used as it had a higher success rate. DNA was extracted from each fungal culture and ITS (internal transcribed spacer 1 and 2 of nuclear ribosomal DNA, ITS1 and ITS4 [31]), LSU (large subunit of nuclear ribosomal DNA, LROR and LR5 [32]) and TEF1α (transcription elongation factor 1, TEF1-983F and TEF1-1567R [33]) was amplified (for PCR protocols see Supplementary Table S1 and Supplementary Table S2). PCR products were purified using ExoSAP-ITTM (Thermo Fisher Scientific; Waltham, MA, USA) and sequenced in both directions using automated Big Dye terminator Sanger sequencing (by Macrogen Inc.; Amsterdam, The Netherlands) using the same primers as in the respective PCRs. Sequences are deposited in GenBank under accession numbers X-X.

### 2.5. Endophyte Identification

Cultures from site III were chosen for DNA barcoding identification because the site showed the largest number of isolated endophytes and it was hypothesised that it could contain the highest total species richness. Morphological identification was not used because of the large sample size and lack of useful morphological structures in many samples such as spores. The ITS sequences were used for identification because they were amplified and sequenced most consistently. Furthermore, the ITS region was also used for the Illumina culture-independent sequencing so the results from the two approaches could be directly compared using the ITS region. The identifications based on ITS were cross checked to LSU and TEF1α sequences when those sequences were available.

Neighbour-joining trees based on p-distance were made for each barcoding region using the software MEGA7: molecular evolutionary genetics analysis across computing platforms [34]. Sequences were edited and trimmed in MEGA7. Then, individual trees were built for each taxonomic class of fungi separately to examine if the same number of operational taxonomic units (OTUs) would be determined. OTUs define individual sequences which are closely related [35]. Clusters were defined as OTUs if their members had at least 99% sequence similarity.

To assign a name to the OTU clusters, the ITS sequences were compared to the UNITE database (https://unite.ut.ee/; accessed on 8 March 2018 to 3 July 2018) [36] and assigned a taxon classification if the percentage identity was in the range of 99–100%. When there were discrepancies for the identification within an OTU cluster, the following steps were taken to allocate the taxonomic name and manage incongruence: (1) evaluate the quality of the sequence and (2) compare levels of percentage identity (only 99–100% was accepted). When identity was lower than 99% the cluster was assigned to the consensus taxonomic class.

### 2.6. Sequence Processing and Community Analyses

Bioinformatic processing of the Illumina sequencing data was undertaken with demultiplexed paired-end reads using the microbiome analysis package Qiime 2, version qiime2-2018.6 [37]. Sequences were denoised, trimmed, joined, chimera were removed and sequences were quality filtered using Dada2 [38] following essentially the “Moving Pictures” tutorial https://docs.qiime2.org/2019.1/tutorials/moving-pictures/ (accessed on 1 February 2019) (Supplementary Data S4). Furthermore, all sequences that were 95% identical to, and had 95% overlap with, a selection of plant ITS sequences were removed from the dataset according to a BLAST search. Classification was performed using the UNITE developers classifier, UNITE Community (2017): UNITE QIIME release. Version 01.12.2017. UNITE Community. The data were not rarefied [39,40] and low frequency clusters were not removed [41].

The data were analysed with the software package R i386 3.4.3 (https://cran.r-project.org/bin/windows/base/old/3.4.3/) (accessed on 1 February 2019). Linear models with the appropriate random effects were fitted and tested against each other using ANOVA. The data followed a normal distribution and the residuals were homogenous and independent. Multiple comparisons were made using Bonferroni-adjusted *p*-values with significance level (*p* ≤ 0.05).

Non-metric multidimensional scaling (NMDS) was performed using the R package vegan [42]. Bray–Curtis distances for binary data were used with 100 iterations and the stress was < 0.05 and thus provided an excellent representation in reduced dimensions. Beta diversity was calculated using the following equations, first for all sites and then for site III specifically. The gamma diversity is the total number of recorded species in the area of interest and alpha diversity is the average number of recorded species across all plants or each plant in site III.

$${\mathrm{Beta}\;\mathrm{diversity}}_{\mathrm{all}\;\mathrm{sites}}=\frac{{\mathrm{gamma}\;\mathrm{diversity}}_{\mathrm{all}\;\mathrm{sites}}\;}{\mathrm{average}\left({\mathrm{alpha}\;\mathrm{diversity}}_{\mathrm{all}\;48\;\mathrm{plants}}\right)}$$

$${\mathrm{Beta}\;\mathrm{diversity}}_{\mathrm{site}\;\mathrm{III}}=\frac{{\mathrm{gamma}\;\mathrm{diversity}}_{\mathrm{site}\;\mathrm{III}}\;}{\mathrm{average}\left({\mathrm{alpha}\;\mathrm{diversity}}_{\mathrm{each}\;\mathrm{plant}\;\mathrm{in}\;\mathrm{site}\;\mathrm{III}}\right)}$$

## 3. Results

### 3.1. OTU Richness Described by Direct Amplicon Sequencing

#### 3.1.1. The OTU Richness and Community Structure of All Sites

**Figure 1:**
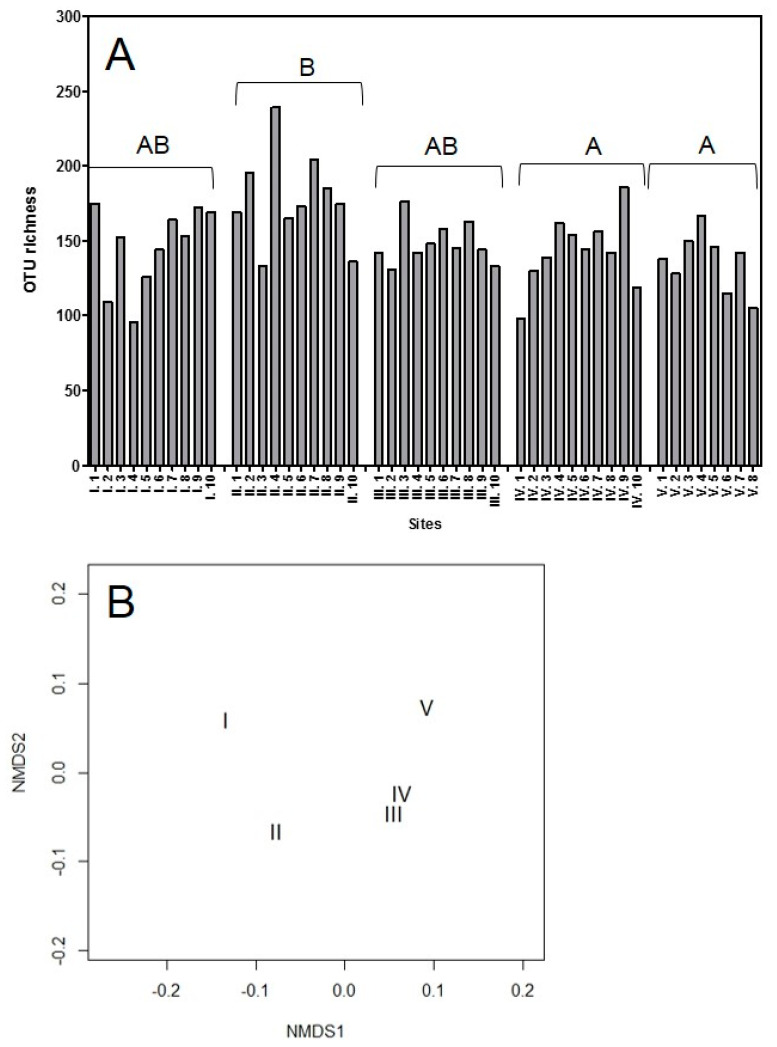
OTU richness determined by direct amplicon sequencing of roots from five sites and community composition. (**A**) Variation in OTU richness across five sites at individual plant level. Capital letters show differences in mean number of OTUs identified per site. Different capital letters represent significant differences; sites sharing the same letter are not significantly different (*p* ≤ 0.05). Created in GraphPad Prism 5. (**B**) Comparison of the community composition between the five sites using non-metric multidimensional scaling, stress was <0.05. Created in R version 3.4.3.

To our knowledge, this is the first time the endophytic communities of individual plants have been studied in grasses because other studies pooled their samples from individual plants before making community assessments. There was a large variation in the number of OTUs identified from individual plants ranging from 96 OTUs (site I, plant 4) to 239 OTUs (site II, plant 4), with a mean of 151 OTUs identified across all 48 plants (Figure 1A). In addition, the five sites showed different fungal community compositions. Across all 48 plants, the beta diversity quantified 4.7 communities which corresponded well with the non-metric multidimensional scaling, which suggested that all five sites had unique communities, with the communities in site III and site IV being the most similar to each other (Figure 1B).

From all five sites, three different kingdoms of organisms were discovered living as endophytes within *Elymus repens* roots, using direct amplicon sequencing, namely the Chromista, Fungi and Rhizaria (Table 1). In total, 715 different fungal OTUs were discovered from the five sites and they belonged to 8 different taxonomic divisions and 31 classes (Table 1).

#### 3.1.2. The OTU Richness and Community Structure of Site III

**Figure 2:**
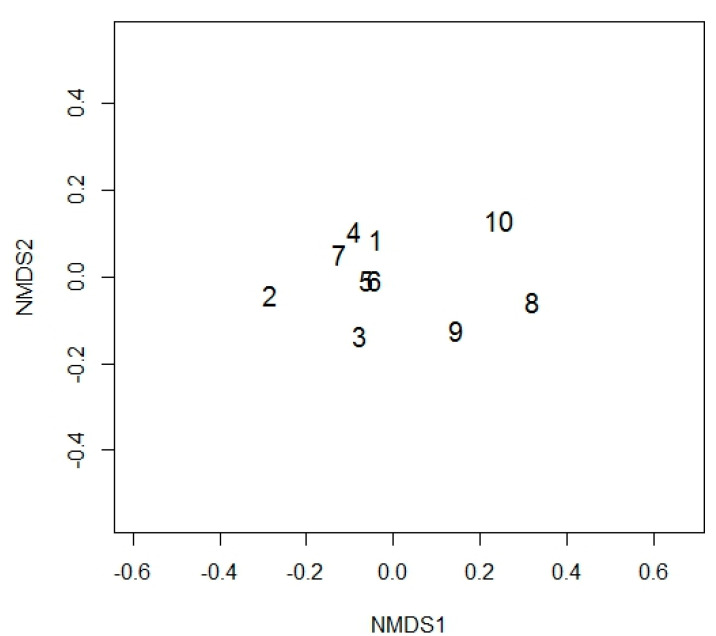
Non-metric multidimensional scaling of the communities found within roots from the ten plants sampled within site III, stress was <0.05. Created in R version 3.4.3.

Our detailed assessment of one site (site III) showed that there was also separation between the community compositions within individual plants. Across all 10 plants, the beta diversity quantified 2.3 communities with separation for these communities apparent in the non-metric multidimensional scaling (Figure 2).

Only fungi were identified from site III and these belonged to 21 different classes (Table 1). A total of 349 different OTUs were identified from this site (Figure 1A) and the average OTU richness per root system was 148. A subset of 48 OTUs could be found widespread in all root systems and they belonged to seven different classes including the Dothideomycetes (16 OTUs), Eurotiomycetes (two OTUs), Leotiomycetes (11 OTUs), Sordariomycetes (13 OTUs), Agaricomycetes (one OTU), Glomeromycetes (one OTU) and Mortierellomycetes (one OTU, Table 2).

### 3.2. Cultured OTU Richness from Site III

#### 3.2.1. Inter-Plant Variation in OTU Richness

**Figure 3:**
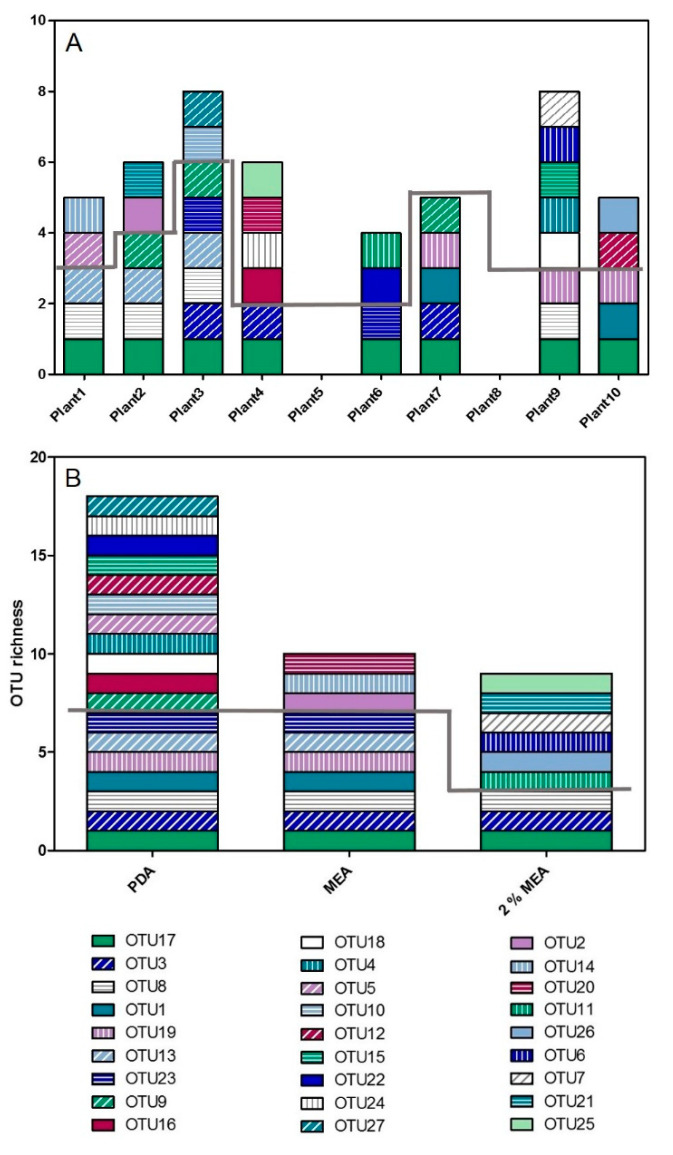
OTU richness cultured from individual plants in site III and media influence on OTU richness. Each colour pattern represents a different OTU and their taxonomic identification can be found in Table 3. (**A**) OTUs isolated from individual plants. The OTUs below the grey line can all be found in more than one plant and the OTUs above the grey line are unique to the specific plant. No endophytes were cultured from plant 5 and no ITS sequence was amplified from the cultures isolated from plant 8. (**B**) OTU richness isolated on three types of media. The OTUs below the grey line can all be isolated on PDA whereas the OTUs above the grey line were only isolated on a specific medium. Created in GraphPad Prism 5.

Cultured endophytes are most often pooled when studies describe the endophyte community of plants, but interestingly there was also a high variation in the isolated endophytes on individual plant level (Figure 3A). Such variation was also seen in the direct sequencing (Figure 2). On average six OTUs were isolated from each plant root system from site III and the combination of OTUs isolated from each of the individual plants was unique (Figure 3A). All plant roots had one OTU in common, identified as *Leptodontidium* sp. (OTU17). The second most dominating OTU was most likely *Ophiosphaerella* sp. (OTU8-Dothideomycetes sp. 7, Supplementary Table S3) isolated from four plant roots, followed by Chaetosphaeriaceae sp. (OTU19), Dothideomycetes sp. 2 (OTU3), *Epicoccum nigrum* (OTU9) and *Periconia* sp. 1 (OTU13) isolated from three plants. Plant 2 and plant 3 had four OTUs in common which was the highest number of shared OTUs.

#### 3.2.2. Media Influence on OTU Richness

Species richness and composition of retrieved endophytes differed among the three types of media. The highest OTU richness was discovered on PDA with 18 OTUs, followed by MEA with 10 OTUs and 2% MEA with 9 OTUs (Figure 3B). Only three OTUs could be discovered by all media, namely Dothideomycetes sp. 2 (OTU3), *Ophiosphaerella* sp. (OTU8) and *Leptodontidium* sp. (OTU17, Figure 3 and Table 3). The media PDA and MEA additionally shared four OTUs which included Chaetosphaeriaceae sp. (OTU19), *Clohesyomyces* sp. (OTU1), Lasiosphaeriaceae sp. (OTU23) and *Periconia* sp. 1 (OTU13). The remaining 20 OTUs were only found on one specific medium and therefore using PDA only would have excluded approximately 50% of the unique OTUs. Unique OTUs were isolated from all plant root systems, except plant 7. The number of unique OTUs in each root system ranged from two (plant 1, 2, 6, 10) to five (plant 9).

## 4. Discussion

### 4.1. Endophyte Community Described by Direct Amplicon Sequencing

Organisms belonging to three kingdoms including Chromista, Fungi and Rhizaria were identified as root endophytes of *Elymus repens* by direct amplicon sequencing of roots. Plant associated organisms are found within the Chromista including plant pathogens belonging to the Oomycetes such as *Phytophthora* sp. causing as examples potato late blight [43] and collar rot of Kauri, *Agathis australis* [44]. The kingdom Rhizaria belongs to the paraphyletic protists [45] and was represented by an OTU within the phylum Cercozoa that was identified from two individual plants of *E. repens*. There are several root endophytic and plant pathogenic Cercozoa [46] including, as examples, *Plasmodiophora brassicae* causing clubroot in crucifers and *Spongospora subterranea* causing potato powdery scab disease [47].

There was a large degree of variation in the OTU richness identified from each root system from the five sites. Across all sites, each plant had an average of 151 OTUs determined by direct amplicon sequencing and an average of 8 isolates were cultured from each plant from a total pool of 715 different OTUs determined by direct amplicon sequencing. The fungal OTUs identified by direct amplicon sequencing from *E. repens* belonged to 31 taxonomic classes (Table 1) from a total of 56 fungal classes recognised in the UNITE database (https://unite.ut.ee/, accessed on 1 February 2019 [36]).

### 4.2. Comparison Between the Cultured and Directly Sequenced Community of Site III

The endophyte community identified by direct amplicon sequencing was much more species rich than the cultured community. A total of 349 OTUs, belonging to 21 classes and six divisions, were identified from site III using amplicon sequencing (Table 1 and Figure 1). In comparison, only 27 OTUs, from four classes belonging to one division, was identified using cultures (Table 3). Using direct amplicon sequencing, it also became clear that all plants hosted endophytes which were not evident or detectable from the culturing technique alone.

It was hypothesised that the most widespread fungal species would also be the ones that were predominantly cultured. A total of 48 OTUs were identified across all plants of site III using amplicon sequencing and, interestingly, only four of these OTUs/species names were shared with the cultured community. The overlapping species included *Ophiosphaerella* sp. and *Periconia* sp. (Dothideomycetes), *Glarea* sp. (Leotiomycetes) and *Gaeummanomyces graminis* (Sordariomycetes). *Ophiosphaerella* sp. and *Periconia* sp. were among some of the species cultured relatively frequently. However, *Glarea* sp. and *Gaeummanomyces graminis* were only isolated once. An additional six species identifications were shared between the two types of methods and included Dothideomycetes sp. 2 and 3 (OTU3 and OTU4—most likely Pleosporales sp., Supplementary Table S3), Chaetosphaeriaceae sp. (OTU19), *Diaporthe* sp. (OTU20), Lasiosphaeriaceae sp. (OTU23), Sordariomycetes sp. 1 (OTU24—most likely *Falciphora* sp.) and Xylariaceae sp. (OTU27).

The endophyte that was cultured from all roots (*Leptodontidium* sp.; OTU17) is surprisingly not on the list of endophytes found in all plants from site III identified by direct amplicon sequencing. The identification of OTU17 was not straightforward (Supplementary Table S3) and if this OTU had been identified as Helotiales sp. then there would have been a match to the 48 OTUs that were present in all ten plants of site III.

Several culturable fungi were found in the amplicon sequencing dataset with examples such as *Alternaria* spp., *Aspergillus* spp., *Trichoderma* spp. and *Verticillium* spp. which were not cultured. This suggests that the cultured endophyte community is a fraction of what could potentially be cultured. In addition, most of the widespread fungi from direct amplicon sequencing were not recovered. Jayawardena et al. [48] suggest that the fast growing fraction is cultured and that these fungi might not represent the most widespread in the community. Some endophytes could be antagonistic to others on isolation media and some could be more sensitive to the surface sterilisation procedure than others.

A limited number of studies have compared the fungal community estimated by direct sequencing with the community estimated by culturing methods in grasses. Yuan et al. [49] found that the cultured community on MEA had a few taxa overlapping with the directly sequenced community from wild rice, *Oryza granulata*. However, Tejesvi et al. [25] did not find any similarities between the cultured community on MEA and PDA, and the directly sequenced community of fungal root endophytes of the wavy hair grass, *Deschampsia flexuosa*. Our study of root endophytes of *E. repens* shows that the cultured endophytes are both a subset of the total community explored with direct amplicon sequencing and that most of the cultured endophyte set do not overlap with the amplicon dataset. The non-existing overlap for the majority of OTUs could reflect errors in the identification process. However, high percent identity scores were used and the same database (UNITE) as well as barcoding region (ITS) were employed. It is possible that the lack of overlap in the two communities is due to the use of different forward primers. For direct amplicon sequencing, fITS7 was used, whereas ITS1 was used for the cultured communities. fITS7 is more specific to fungi and was used in the amplicon sequencing to reduce the co-amplification of plant DNA; in the cultured fungal sequencing this was not an issue. In both the studies by Tejesvi et al. [25] and Yuan et al. [49] different primer pairs were employed for culture-dependent and -independent identification and the studies came to very different conclusions, no overlap and some taxa overlapping, respectively. Dissanayake et al. [50] also used different primer pairs and found 53% species composition overlap when they studied the endophyte communities of stems of grapevine, *Vitis vinifera*. They identified their cultured community using nine different barcoding regions and perhaps emphasis should be put on good identification of the cultured community. Studies will often explain that they assigned a name to their OTU based on the percent similarity obtained from the top hit of a database. Thus, another possibility for the difference in community overlap is the choice of percent similarity used when assigning names to OTUs. Tejesvi et al. [25] used 95% homology and had no overlap, Dissanayake et al. [50] used 90% for genera and 97% and above for species with 53% overlap and Yuan et al. [49] used 99% or above and found little overlap. Based on this, to get good correlation between cultured community and directly sequenced community using a middle ground might be necessary.

Nilsson et al. [51] showed that the intraspecific ITS variability is dependent on species so there is no common yardstick for the variation expected in a fungal genus, family or any higher taxon. There is no one universally applicable percentage cut off value. However, 3% has become widely used [52]. Gazis et al. [53] and Luo et al. [22] compared diversity determined by 1% and 3% clustering criteria. Luo et al. [22] examined the root endophyte community from rosette grass, *Dichanthelium acuminatum*; switchgrass, *Panicum virgatum*; and pitch pine, *Pinus rigida* and found that the two cut off values resulted in similar community structure estimations. In contrast, Gazis et al. [53] studied three species complexes within the Sordariomycetes and found that increasing the percent similarity cut off value increased the number of OTUs. The intraspecific variation within the ITS region from fungi within the INSD database was examined by Nilsson et al. [51] and they found species with very low intraspecific variation 0.2% (*Aspergillus fumigatus* and *Candida albicans*) and species with very high variation 24.2% (*Xylaria hypoxylon*). The big difference in intraspecific variation between species might explain why Gazis et al. [53] and Luo et al. [22] had conflicting results. Across all the examined species, Nilsson et al. [51], found that the majority of species had intraspecific variability of 0–1%, and thus a 1% cut off value was adopted in this study. It is also possible that the time lapse between querying UNITE about individual sequences made a difference to identification. However, only approximately three months passed between identifying the cultures and the sequenced community. It is therefore most probable that the pattern is real.

### 4.3. The Influence of Media on OTU Richness

This is the first evaluation of how the most commonly used media for isolation of endophytes of grasses can influence the isolation success. The majority of OTUs were discovered on PDA (18 OTUs) followed by MEA (10) and 2% MEA (9). The overall difference in these three media is the sugar source and the strength. PDA is composed of the monosaccharide dextrose and potato extract while MEA has dextrin, the disaccharide maltose and vegetable peptone [54]. A few studies of endophytes of grasses have isolated endophytes on several media but they do not discuss their influence on endophyte diversity [24,25,55,56]. Verma et al. [18] isolated endophytes from the neem tree on four different media and found that the maximum number of endophytes was recovered from PDA.

The only known previously successful biocontrol agent, *Epicoccum* ssp. (OTU9 and OTU10) was only isolated on PDA. *Gaeumannomyces graminis* (OTU22), a known pathogen of barley and wheat [57], was only isolated on PDA. In contrast, *Ophiosphaerella* spp. (OTU11, OTU12 and OTU3) was isolated on all three media and has also been reported as a pathogen of a range of grasses. Known pathogens are often found as endophytes within non-symptomatic plants [58,59,60], which highlights the knowledge gap of the functional roles of endophytes and the abiotic as well as biotic cues that might change those roles.

## 5. Conclusions

This study illustrates many of the issues at the core of endophyte discovery and community description. PDA medium recorded the highest species richness but also excluded many rare species. Only a fraction of those endophytes that could potentially be isolated were cultured and did not represent the most widespread species. Furthermore, large variation in the fungal species richness estimates highlights the high heterogeneity at both plant and site level. Despite the attention received, the field is still some way off in developing a satisfactory methodology with the desired outcomes. Our results indicate that a combination of culture-dependent and -independent methods is the best approach for estimating the root fungal species richness.

## Figures and Tables

**Table 1:** Overview of the different classes of endophytes identified from *Elymus repens* roots from all sites. It lists the number of plants that contained each fungal class and in how many sites the class was present.

Kingdom	Division	Class	Number of Plants	Site				
				I	II	III	IV	V
Chromista	–	–	2		×			
Fungi	Ascomycota	Archaeorhizomycetes	12	×	×	×	×	×
		Dothideomycetes	48	×	×	×	×	×
		Eurotiomycetes	48	×	×	×	×	×
		Lecanoromycetes	13	×	×	×	×	×
		Leotiomycetes	48	×	×	×	×	×
		Orbiliomycetes	16	×	×	×	×	×
		Pezizomycetes	34	×	×	×	×	×
		Saccharomycetes	43	×	×	×	×	×
		Sordariomycetes	48	×	×	×	×	×
		Taphrinomycetes	1		×			
		Xylonomycetes	2		×			
	Basidiomycota	Agaricomycetes	48	×	×	×	×	×
		Agaricostilbomycetes	1				×	
		Exobasidiomycetes	1				×	
		Malasseziomycetes	32	×	×	×	×	×
		Microbotryomycetes	26	×	×	×	×	×
		Pucciniomycetes	4		×			
		Tremellomycetes	47	×	×	×	×	×
		Tritirachiomycetes	1	×				
		Ustilaginomycetes	17	×	×	×	×	×
		Wallemiomycetes	7	×		×	×	×
	Chytridiomycota	Rhizophydiomycetes	1	×				
		Spizellomycetes	4	×	×		×	×
	Glomeromycotina	Archaeosporomycetes	6	×	×			
		Glomeromycetes	48	×	×	×	×	×
		Paraglomeromycetes	12	×	×	×	×	×
	Mortierellomycota	Mortierellomycetes	48	×	×	×	×	×
	Mucoromycota	Endogonomycetes	13	×	×	×	×	×
		Mucoromycetes	32	×	×	×	×	×
	Olpidiomycota	Olpidiomycetes	3		×	×		
	Rozellomycota	–	5	×	×			
Rhizaria	Cercozoa	–	2	×			×	

**Table 2:** The 48 OTUs that were present in all plants from site III and their identification. Names of organisms are given according to Species Fungorum (http://www.indexfungorum.org/; accessed on 1 February 2019).

Class	Species	Class	Species
–	Fungi sp. 1	Leotiomycetes	*Articulospora* sp.
–	Fungi sp. 2		*Glarea* sp.
–	Ascomycota sp.		*Hymenoscyphus* sp.
Dothideomycetes	Dothideomycetes sp.		*Tetracladium* sp.
	Capnodiales sp.		*Tetracladium marchalianum*
	Pleosporales sp.		*Tricladium splendens*
	*Xenopyrenochaetopsis pratorum*		*Rhexocercosporidium panacis*
	Didymellaceae sp.		*Microscypha* sp.
	*Neoascochyta graminicola*	Sordariomycetes	Sordariomycetes sp.
	Didymosphaeriaceae sp.		*Codinaea acaciae*
	*Stagonospora pseudovitensis*		*Pseudolachnella* sp.
	Melanommataceae sp.		*Gibellulopsis nigrescens*
	*Periconia* sp.		*Dactylonectria macrodidyma*
	*Ophiosphaerella* sp.		*Gaeumannomyces graminis*
	*Phaeosphaeria triglochinicola*		*Slopeiomyces cylindrosporus*
	Phaeosphaeriaceae sp.		*Myrmecridium* sp.
	*Alternaria* sp.		*Pleotrichocladium opacum*
	*Alternaria hordeicola*		*Schizothecium glutinans*
	*Drechslera* sp.		*Microdochium* sp.
Eurotiomycetes	*Exophiala* sp.		*Microdochium bolleyi*
	*Aspergillus sydowii*		*Microdochium phragmitis*
Leotiomycetes	Helotiales sp.	Agaricomycetes	Agaricomycetes sp.
	Helotiaceae sp. 1	Glomeromycetes	Glomeraceae sp.
	Helotiaceae sp. 2	Mortierellomycetes	*Mortierella exigua*

**Table 3:** Taxonomic identification of OTUs from the cultured endophytes from site III using ITS sequences. The sequences were compared using BLAST through the UNITE database. For a more detailed examination of the identification see Supplementary Table S3. 12 cultures could not be identified because no DNA was extracted and for 27 cultures no ITS sequence was amplified so these cultures were categorised as individual OTUs. Names of organisms are given according to Species Fungorum (http://www.indexfungorum.org/; accessed on 8 March 2018 to 3 July 2018).

OTU	Sequence(s)	Class	Identification
1	4	Dothideomycetes	*Clohesyomyces* sp.
2	1		Dothideomycetes sp. 1
3	8		Dothideomycetes sp. 2
4	1		Dothideomycetes sp. 3
5	1		Dothideomycetes sp. 4
6	1		Dothideomycetes sp. 5
7	1		Dothideomycetes sp. 6
8	15		Dothideomycetes sp. 7
9	6		*Epicoccum nigrum*
10	1		*Epicoccum* sp.
11	2		*Ophiosphaerella korrea*
12	1		*Ophiosphaerella* sp. 1
13	4		*Periconia* sp. 1
14	1		*Periconia* sp. 2
15	1		Pleosporaceae sp.
16	2	Leotiomycetes	*Glarea* sp.
17	34		*Leptodontidium* sp.
18	2	Pezizomycetes	*Pyronema domesticum*
19	7	Sordariomycetes	Chaetosphaeriaceae sp.
20	1		*Diaporthe sp.*
21	2		*Falciphora* sp.
22	1		*Gaeumannomyces graminis*
23	2		Lasiosphaeriaceae sp.
24	1		Sordariomycetes sp. 1
25	1		Sordariomycetes sp. 2
26	2		Sordariomycetes sp. 3
27	1		Xylariaceae sp.
28–54	27		Fungus sp. 28–54
55–66	12		Fungus sp. 55–66

## Data Availability

The DNA datasets generated during the current study are in the process of being available from the GenBank repository (NCBI). The code used in Qiime2 is available in Supplementary Data S4.

## Supplementary Materials

The following are available online at https://www.mdpi.com/article/10.3390/jof7060466/s1, Table S1: Primers used to amplify DNA from fungal endophytes, the primer sequence and the reference. Table S2: PCR cycles used for the individual primers. Temperature [°C] and time [min]. Table S3: Taxonomic identification of OTUs from the cultured endophytes from site III using ITS sequences. Data S4: Code for bioinformatics analysis of demultiplexed paired-end reads in Qiime2.
