# Supplementary material for: Hidden Fungi: Combining Culture-Dependent and -Independent DNA Barcoding Reveals Inter-Plant Variation in Species Richness of Endophytic Root Fungi in Elymus repens
Source: J Fungi (Basel). 2021 Jun 9;7(6):466. doi: 10.3390/jof7060466 (PMC8226481; doi:10.3390/jof7060466)
Supplement: Supplementary file 1 [file jof-07-00466-s001.zip › jof-1232529-supplementary.pdf]

Supplementary Table S1

Table S1. Primers used to amplify DNA from fungal endophytes, the primer sequence and the reference.

Locus	Primer	Primer sequence	Reference
ITS	ITS1	5' TCCGTAGGTGAACCTGCGG 3'	White *et al.* 1990
	ITS4	5' TCCTCCGCTTATTGATATGC 3'	White *et al.* 1990
	fITS7	5' GTGARTCATCGAATCTTTG 3'	Ihrmark *et al.* 2012
LSU	LROR	5' ACCCGCTGAACTTAAGC 3'	Stielow *et al.* 2015
			Johnson and Vilgalys 1998
	LR5	5' TCCTGAGGGAAACTTCG 3'	Stielow *et al.* 2015
TEF1α	EF1-983F	5' ACHGTRCCRATACCACCSATCTT 3'	Rehner and Buckley 2005
	EF1-1567R	5' GCYCCYGGHCAYCGTGAYTTYAT 3'	Rehner and Buckley 2005

Supplementary Table S2

Table S2. PCR cycles used for the individual primers. Temperature [˚C] and time [minutes].

Primer	TEF1α		ITS		LSU	
	Temperature	Time	Temperature	Time	Temperature	Time
Premelt	94	1.30	94	1.30	94	1.30
Denature	94	0.45	94	1.30	94	0.30
Anneal	61	1.00	58	1.00	53	1.00
Extension	72	1.00	72	1.00	72	1.00
Final extension	72	7.00	72	7.00	72	7.00
No. cycles	30	-	30	-	30	-

Supplementary Table S3

Table S3. Taxonomic identification of OTUs from the cultured endophytes from site III using ITS sequences. The sequences were blasted through the UNITE database. In the first section of the table the number of sequences used to form the OTU, the class and the final identification are listed. In the next two sections the top hit, the next best hit as well as the percentage identity and the accession numbers are listed. The next best hit is listed when the top hit was below 99 % identity. The next best hit is defined as the hit that gave more resolution than the top hit. 12 cultures could not be identified because no DNA was extracted and for 27 cultures no ITS sequence was amplified so these cultures were grouped into individual OTUs. Names of organisms are given according to Species Fungorum (http://www.indexfungorum.org/).

OTU	Sequence(s)	Class	Final identification	Top hit	Identity %	Accession number	Next best hit[A]	Identity %	Accession number
1	4	Dothideomycetes	*Clohesyomyces* sp.	*Clohesyomyces* sp.	99	KT269257	-	-	-
2	1		Dothideomycetes sp. 1	Fungi	96	KT203006	*Ophiosphaerella* sp.	96	KT269297
3	8		Dothideomycetes sp. 2	Pleosporales sp.	98	GQ996144	*Camposporium cambrense*	96	KY853428
4	1		Dothideomycetes sp. 3	Pleosporales sp.	79	EU490184	*Phaeosphaeria* sp.	79	EF590323
5	1		Dothideomycetes sp. 4	*Clohesyomyces* sp.	90	JQ435795	*Clohesyomyces* sp.	94	KT269257
6	1		Dothideomycetes sp. 5	Fungi	87	UDB028789	*Abrothallus suecicus*	88	UDB018278
7	1		Dothideomycetes sp. 6	*Clohesyomyces* sp	94	JQ435795	*Clohesyomyces aquaticus*	91	JX276948
8	15		Dothideomycetes sp. 7	Fungi	99	KT203006<br>KC007324<br>KT269297	*Ophiosphaerella* sp.	99	KT269297
9	6		*Epicoccum nigrum*	*Epicoccum nigrum*	100	KX426949<br>MF509753	-	-	-
10	1		*Epicoccum* sp.	*Epicoccum* sp.	99	MF788189	-	-	-
11	2		*Ophiosphaerella korrea*	*Ophiosphaerella korrea*	99	AF486626	-	-	-
12	1		*Ophiosphaerella* sp. 1	*Ophiosphaerella* sp	99	KT692575	-	-	-
13	4		*Periconia* sp. 1	*Periconia* sp.	99	HG936263	-	-	-
14	1		*Periconia* sp. 2	*Periconia* sp.	99	KT269450	-	-	-
15	1		Pleosporaceae sp.	Pleosporaceae sp.	100	EU754971	-	-	-
16	2	Leotiomycetes	*Glarea* sp.	*Glarea* sp.	99	KT268823	-	-	-
17	34		*Leptodontidium* sp.	*Leptodontidium* sp. (17)[B]<br>*Cadophora orchidicola* (1)<br>Lasiosphaeriaceae sp. (7)<br>Helotiales sp. (9)	100<br>99<br>100<br>100	KU886584<br>KX440156<br>KY430565<br>HG936155	*Cadophora* sp.<br>*Leptodontidium* sp.<br>*Cadophora* sp.<br>Lasiosphaeriaceae sp.	100<br>99<br>100<br>99	KT269226<br>KF428333<br>KT269262<br>KY430518
18	2	Pezizomycetes	*Pyronema domesticum*	*Pyronema domesticum*	100	HQ115722	-	-	-
19	7	Sordariomycetes	Chaetosphaeriaceae sp.	Chaetosphaeriaceae sp.	99	GU327452	-	-	-
20	1		*Diaporthe sp.*	*Diaporthe sp.*	100	KP984752	-	-	-
21	2		*Falciphora* sp.	*Falciphora* sp.	99	HG937137	-	-	-
22	1		*Gaeumannomyces graminis*	*Gaeumannomyces graminis*	99	KT819302	-	-	-
23	2		Lasiosphaeriaceae sp.	Lasiosphaeriaceae sp.	100	KY430501<br>KY430565	-	-	-
24	1		Sordariomycetes sp. 1	*Falciphora* sp.	98	KX306546	*Falciphora oryzae*	94	EU63699
25	1		Sordariomycetes sp. 2	*Dictyochaetopsis gonytrichoides*	97	AF178556	*Dictyochaeta simplex*	95	AF178559

26	2	Sordariomycetes sp. 3	-[C]	-	-	-	-	-
27	1	Xylariaceae sp.	Xylariaceae sp.	100	KX067819	-	-	-
28-54	27[D]	Fungus sp.						
55-66	12[E]	Fungus sp.						

[A] This column represents the next best identification that gives a better resolution compared to the top hit which had a lower identity than 99 %. Sometimes the identity percentage is the same or higher than in the top hit column because the sequences are scored on multiple criteria, not listed here, which also influences their ranking. As an example, if the top hit is an order then a lower rank is listed.

[B] In brackets are the number of sequences that contributed to the identification. The majority of sequences (27 out of 34) put this OTU in the Leotiomycetes and the majority was followed for the final naming of this OTU.

[C] The ITS sequences forming this OTU were contaminated with *Penicillium sp.* therefor the LSU sequences were trusted and they grouped this OTU to the Sordariomycetes.

[D] For 27 cultures DNA was extracted but not amplified using the ITS primers. [E] For 12 cultures DNA was not successfully extracted.

Supplementary data S4

Code for bioinformatics analysis of demultiplexed paired-end reads in Qiime2.

```
# Directory overview, folders are in bold
      # 2NGS
            #data
                  # 00.RawData
                  # manifest_raw
                  # Elymus_features
                  # UNITE_classifier


# Activate qiime
source activate qiime2-2018.6

# Import data into Qiime2
qiime tools import \
  --type 'SampleData[PairedEndSequencesWithQuality]' \
  --input-path manifest_raw \
  --output-path paired-end-demux.qza \
  --source-format PairedEndFastqManifestPhred33

# Generate summary of demultiplexed data, determine how many
# sequences were obtained per sample, and also get a summary
# of the distribution of sequence qualities at each position in
# your sequence data
qiime demux summarize \
  --i-data paired-end-demux.qza \
  --o-visualization paired-end-demux.qzv

# Remove the primer sequence from the raw sequences
qiime cutadapt trim-paired \
  --i-demultiplexed-sequences paired-end-demux.qza \
  --p-front-f GTGARTCATCGAATCTTTG \
  --p-front-r TCCTCCGCTTATTGATATGC \
  --p-error-rate 0.1 \
  --p-cores 6 \
  --o-trimmed-sequences trimmed-seqs.qza

# Dada2: denoising, trimming, chimera removal
qiime dada2 denoise-paired \
  --i-demultiplexed-seqs trimmed-seqs.qza \
  --p-trim-left-f 0 \
  --p-trim-left-r 0 \
  --p-trunc-len-f 222 \
  --p-trunc-len-r 220 \
  --o-table table_trimdada.qza \
  --o-representative-sequences rep-seqs_trimdada.qza \
  --o-denoising-stats denoising-stats_trimdada.qza

# Create FeatureTable and FeatureData summaries
qiime feature-table summarize \
  --i-table table_trimdada.qza \
  --o-visualization table_trimdada.qzv \


# Created a folder, Elymus_features, with the following plant ITS
# fasta files:

# Elymus_5.8S.fasta FJ793076.1
```

```
# Elymus_ITS1.fasta FJ793076.1
# Elymus_ITS2.fasta FJ793076.1
# Elymus_antiquus.fasta AY740818.1
# Elymus_caninus.fasta AY740897.1
# Elymus_fullsequence.fasta FJ793076.1
# Elymus_lanceolatus.fasta EF396961.1
# Elymus_sibiricus.fasta EF396962.1
# Elymus_tauri.fasta EF014244.1
# Elymus_tauri_short.fasta EU617238.1
# Agrostis_gigantea KY872905.1
# Agrostis capillaris KX872899.1
# Agrostis stolonifera KX872911.1
# Agrostis hallii  KX872907.1
# Elymus tangutorum KF905148.1
# Elymus repens FJ793087.1
# Hordeum brachyantherum MG215969.1
# Elymus atratus KJ526331.1
# Kengyilia gobicola JF976721.1
# Agropyron krylovianum KJ561240.1
# Hordeum roshevitzii KU513502.1
# Elymus_virginicus.fasta MG215649.1
# Elymus_glaberrimus.fasta AY740844.1
# Elymus_stipifolia.fasta EU617049.1
# Prunus_domestica.fasta KX166465.1
# Prunus_spinosa.fasta KX167489.1
# Prunus_bokhariensis.fasta GQ179665.1
# Prunus_armeniaca.fasta JF978104.1
# Prunus_sibirica.fasta AF318739.1
# Elymus_nutansxkengyilia.fasta JQ670990.1
# Trebouxia_impressa.fasta KX181276.1

# Combined the plant ITS sequences using cat
cat Elymus_5.8S.fasta Elymus_ITS1.fasta Elymus_ITS2.fasta
Elymus_antiquus.fasta Elymus_caninus.fasta Elymus_fullsequence.fasta
Elymus_lanceolatus.fasta Elymus_sibiricus.fasta Elymus_tauri.fasta
Elymus_tauri_short.fasta Agrostis_gigantea.fasta
Agrostis_capillairs.fasta Agrostis_stolonifera.fasta
Agrostis_hallii.fasta Elymus_tangutorum.fasta Elymus_repens.fasta
Hordeum_brachyantherum.fasta Elymus_atratus.fasta
Kengyilia_gobicola.fasta Agropyron_krylovianum.fasta
Hordeum_roshevitzii.fasta Elymus_virginicus.fasta
Elymus_glaberrimus.fasta Elymus_stipifolia.fasta
Prunus_domestica.fasta Prunus_spinosa.fasta Prunus_bokhariensis.fasta
Prunus_armeniaca.fasta Prunus_sibirica.fasta
Elymus_nutansxkengyilia.fasta Trebouxia_impressa.fasta >
Plant_features.fasta

# Imported the combined Plant_features into Qiime2 as .qza
qiime tools import \
  --input-path Plant_features.fasta \
  --output-path Plant_features.qza \
  --type 'FeatureData[Sequence]'

# Excluded different plant sequences with 95 % identity to sequences #
within Plant_features.qza
# Removed anything that has a match with at least 95 % identity over
at least 95 % of the sequence length

qiime quality-control exclude-seqs \
  --i-query-sequences rep-seqs_trimdada.qza \
  --i-reference-sequences Elymus_sequences/Plant_features.qza \
```

```
  --p-method blast \
  --p-perc-identity 0.95 \
  --p-perc-query-aligned 0.95 \
  --o-sequence-hits hits95_trimdada.qza \
  --o-sequence-misses misses95_trimdada.qza

qiime feature-table filter-features \
  --i-table table_trimdada.qza \
  --m-metadata-file hits95_trimdada.qza \
  --o-filtered-table no-Plant95_trimdada-table.qza \
  --p-exclude-ids

#Visualise no-Plant table
qiime feature-table summarize \
  --i-table no-Plant95_trimdada-table.qza \
  --o-visualization no-Plant95_trimdada-table.qzv \
  --m-sample-metadata-file metadata2019-2.txt

# List of blastable miss sequences
qiime feature-table tabulate-seqs \
  --i-data misses95_trimdada.qza \
  --o-visualization misses95_trimdada.qzv

# Classifier training (tutorial
https://github.com/gregcaporaso/2017.06.23-q2-fungal-tutorial)
# Obtaining and importing reference data sets (from UNITE) to the
# folder UNITE_classifier
https://files.plutof.ut.ee/doi/0A/0B/0A0B25526F599E87A1E8D7C612D23AF72
05F0239978CBD9C491767A0C1D237CC.zip
# importet files from the developer folder
      # sh_refs_qiime_ver7_97_01.12.2017_dev.fasta
      # sh_refs_qiime_ver7_99_01.12.2017_dev.fasta
      # sh_taxonomy_qiime_ver7_97_01.12.2017_dev.txt
      # sh_ taxonomy _qiime_ver7_99_01.12.2017_dev.txt
      # sh_refs_qiime_ver7_dynamic_01.12.2017_dev.fasta
      # sh_ taxonomy _qiime_ver7_dynamic_01.12.2017_dev.txt

# qiime tools import \
 --type FeatureData[Sequence] \
 --input-path sh_refs_qiime_ver7_99_01.12.2017_dev.fasta \
 --output-path unite-ver7-99-seqs-01.12.2017_dev.qza

# Train classifier
qiime feature-classifier fit-classifier-naive-bayes \
 --i-reference-reads unite-dynamic-seqs.qza \
 --i-reference-taxonomy unite-dynamic-reftax.qza \
 --o-classifier unite-dynamic-classifier.qza

# Taxonomy at 95 %
# Renaming two files
mv misses95_trimdada.qza rep-seqs95_trimdada.qza
mv no-Plant95_trimdada-table.qza table95_trimdada.qza

# Using classifier for taxonomic analysis
qiime feature-classifier classify-sklearn \
  --i-classifier UNITE_classifier/unite-dynamic-classifier.qza \
  --i-reads rep-seqs95_trimdada.qza \
  --o-classification taxonomy95_trimdada.qza

qiime metadata tabulate \
  --m-input-file taxonomy95_trimdada.qza \
```

```
  --o-visualization taxonomy95_trimdada.qzv

qiime taxa barplot \
  --i-table table95_trimdada.qza \
  --i-taxonomy taxonomy95_trimdada.qza \
  --m-metadata-file metadata2019-2.txt \
  --o-visualization taxa-bar-plots95_trimdada.qzv
```
